# Supplementary material for: Topological characteristics of international business cycle synchronization: A network analysis of the BRI economies
Source: PLoS One. 2022 Jun 28;17(6):e0270333. doi: 10.1371/journal.pone.0270333 (PMC9239471; doi:10.1371/journal.pone.0270333)
Supplement: S1 Appendix — This document contains additional details on the 53 sample BRI economies. (PDF) [file pone.0270333.s001.pdf]

S1 Appendix. 53 sample BRI economies.

ID	NAME	ISO	Geographic Regions	ID	NAME	ISO	Geographic Regions
1	China, P.R.	CHN	East Asia	28	Iraq	IRQ	West Asia & North Africa
2	Mongolia	MNG	East Asia	29	Azerbaijan	AZE	West Asia & North Africa
3	Russia	RUS	Central & Eastern Europe	30	Georgia	GEO	West Asia & North Africa
4	Singapore	SGP	Southeast Asia	31	Armenia	ARM	West Asia & North Africa
5	Indonesia	IDN	Southeast Asia	32	Poland	POL	Central & Eastern Europe
6	Malaysia	MYS	Southeast Asia	33	Albania	ALB	Central & Eastern Europe
7	Thailand	THA	Southeast Asia	34	Estonia	EST	Central & Eastern Europe
8	Vietnam	VNM	Southeast Asia	35	Lithuania	LTU	Central & Eastern Europe
9	Philippines	PHL	Southeast Asia	36	Slovenia	SVN	Central & Eastern Europe
10	Cambodia	KHM	Southeast Asia	37	Bulgaria	BGR	Central & Eastern Europe
11	Brunei	BRN	Southeast Asia	38	Czech Republic	CZE	Central & Eastern Europe
12	India	IND	South Asia	39	Hungary	HUN	Central & Eastern Europe
13	Pakistan	PAK	South Asia	40	Republic of Macedonia	MKD	Central & Eastern Europe
14	Sri Lanka	LKA	South Asia	41	Serbia	SRB	Central & Eastern Europe
15	Bangladesh	BGD	South Asia	42	Romania	ROU	Central & Eastern Europe
16	Nepal	NPL	South Asia	43	Slovakia	SVK	Central & Eastern Europe
17	Maldives	MDV	South Asia	44	Croatia	HRV	Central & Eastern Europe
18	Bhutan	BTN	South Asia	45	Latvia	LVA	Central & Eastern Europe
19	United Arab Emirates	ARE	West Asia & North Africa	46	Ukraine	UKR	Central & Eastern Europe
20	Kuwait	KWT	West Asia & North Africa	47	Belarus	BLR	Central & Eastern Europe
21	Turkey	TUR	West Asia & North Africa	48	Moldova	MDA	Central & Eastern Europe
22	Qatar	QAT	West Asia & North Africa	49	Kazakhstan	KAZ	Central Asia
23	Saudi Arabia	SAU	West Asia & North Africa	50	Kyrgyzstan	KGZ	Central Asia
24	Israel	ISR	West Asia & North Africa	51	Turkmenistan	TKM	Central Asia
25	Yemen	YEM	West Asia & North Africa	52	Tajikistan	TJK	Central Asia
26	Egypt	EGY	West Asia & North Africa	53	Uzbekistan	UZB	Central Asia
27	Jordan	JOR	West Asia & North Africa				
