# Supplementary material for: Topological characteristics of international business cycle synchronization: A network analysis of the BRI economies
Source: PLoS One. 2022 Jun 28;17(6):e0270333. doi: 10.1371/journal.pone.0270333 (PMC9239471; doi:10.1371/journal.pone.0270333)
Supplement: Attachment — Submitted filename: PONE-D-21-31756_reviewers comments.R1.pdf [file pone.0270333.s003.pdf]

PLOS ONE

Topological Characteristics of International Business Cycle Synchronization: A Network Analysis of the BRI Economies

--Manuscript Draft--

**Manuscript Number:**	PONE-D-21-31756
**Article Type:**	Research Article
**Full Title:**	Topological Characteristics of International Business Cycle Synchronization: A Network Analysis of the BRI Economies
**Short Title:**	Topological Characteristics of International Business Cycle Synchronization
**Corresponding Author:**	Mai Sichao, Ph.D.<br>Nanchang Hangkong University<br>Nanchang, Jiangxi CHINA
**Keywords:**	Belt and Road Initiative (BRI); Business Cycle Synchronization Network (BCSN); Quasi-correlation Coefficient; Social Network Analysis (SNA); Topological Characteristics.
**Abstract:**	With the support of social network analysis (SNA), this paper focuses on the topological characteristics of the business cycle synchronization network (BCSN) from overall and individual aspect, which are measured by quasi-correlation coefficient between Belt and Road Initiative (BRI) economies from 2000 to 2019. The research result are as follows: First, although there is an overall fluctuation decline in the density and efficiency of BCSN, the BRI strengthen the business cycle synchr2onization relationship; Second, the BCSN presents a topological feature of “small world” which its Topological evolution finally appears to be self-stable; Third, the four subgroups of BCSN show a distribution characteristic of large dispersion and small agglomeration, and BSCN is finally dominated by the two-way overflow groups and broker groups; Fourth, the influence of a country in BCSN don’t fully match its intermediary role, individual characteristics between countries and regions are different, and the level of individual characteristics in regions among East Asia, Central Asia and Central and Eastern Europe are relatively higher; Fifth, China's node strength, centrality and coreness have all increased, and China finally occupy the core position of BCSN, but its role as a hub still needs to be further strengthened.
**Order of Authors:**	Qiu ZhiPing, Ph.D.
	Mai Sichao
**Additional Information:**	
**Question**	**Response**
**Financial Disclosure**<br><br>Enter a financial disclosure statement that describes the sources of funding for the work included in this submission. Review the submission guidelines for detailed requirements. View published research articles from *PLOS ONE* for specific examples.<br><br>This statement is required for submission and **will appear in the published article** if the submission is accepted. Please make sure it is accurate.	The author(s) received no specific funding for this work.

Powered by Editorial Manager® and ProduXion Manager® from Aries Systems Corporation

**Unfunded studies**<br>Enter: *The author(s) received no specific funding for this work.*<br><br>**Funded studies**<br>Enter a statement with the following details:<br>• Initials of the authors who received each award<br>• Grant numbers awarded to each author<br>• The full name of each funder<br>• URL of each funder website<br>• Did the sponsors or funders play any role in the study design, data collection and analysis, decision to publish, or preparation of the manuscript?<br>• **NO** - Include this sentence at the end of your statement: *The funders had no role in study design, data collection and analysis, decision to publish, or preparation of the manuscript.*<br>• **YES** - Specify the role(s) played.<br><br>* typeset	
**Competing Interests**<br><br>Use the instructions below to enter a competing interest statement for this submission. On behalf of all authors, disclose any competing interests that could be perceived to bias this work—acknowledging all financial support and any other relevant financial or non-financial competing interests.<br><br>This statement is required for submission and **will appear in the published article** if the submission is accepted. Please make sure it is accurate and that any funding sources listed in your Funding Information later in the submission form are also declared in your Financial Disclosure statement.<br><br>View published research articles from *PLOS ONE* for specific examples.	The authors have declared that no competing interests exist.

Powered by Editorial Manager® and ProduXion Manager® from Aries Systems Corporation

**NO authors have competing interests**<br><br>Enter: *The authors have declared that no competing interests exist.*<br><br>**Authors with competing interests**<br><br>Enter competing interest details beginning with this statement:<br><br>*I have read the journal's policy and the authors of this manuscript have the following competing interests: [insert competing interests here]*<br><br>* typeset	
**Ethics Statement**<br><br>Enter an ethics statement for this submission. This statement is required if the study involved:<br><br>• Human participants<br>• Human specimens or tissue<br>• Vertebrate animals or cephalopods<br>• Vertebrate embryos or tissues<br>• Field research<br><br>Write "N/A" if the submission does not require an ethics statement.<br><br>General guidance is provided below. Consult the submission guidelines for detailed instructions. **Make sure that all information entered here is included in the Methods section of the manuscript.**	N/A

**Format for specific study types**<br><br>**Human Subject Research (involving human participants and/or tissue)**<br>• Give the name of the institutional review board or ethics committee that approved the study<br>• Include the approval number and/or a statement indicating approval of this research<br>• Indicate the form of consent obtained (written/oral) or the reason that consent was not obtained (e.g. the data were analyzed anonymously)<br><br>**Animal Research (involving vertebrate animals, embryos or tissues)**<br>• Provide the name of the Institutional Animal Care and Use Committee (IACUC) or other relevant ethics board that reviewed the study protocol, and indicate whether they approved this research or granted a formal waiver of ethical approval<br>• Include an approval number if one was obtained<br>• If the study involved *non-human primates*, add *additional details* about animal welfare and steps taken to ameliorate suffering<br>• If anesthesia, euthanasia, or any kind of animal sacrifice is part of the study, include briefly which substances and/or methods were applied<br><br>**Field Research**<br><br>Include the following details if this study involves the collection of plant, animal, or other materials from a natural setting:<br>• Field permit number<br>• Name of the institution or relevant body that granted permission	
**Data Availability**<br><br>Authors are required to make all data underlying the findings described fully available, without restriction, and from the time of publication. PLOS allows rare exceptions to address legal and ethical concerns. See the PLOS Data Policy and FAQ for detailed information.	No - some restrictions will apply

Powered by Editorial Manager® and ProduXion Manager® from Aries Systems Corporation

A Data Availability Statement describing where the data can be found is required at submission. Your answers to this question constitute the Data Availability Statement and **will be published in the article**, if accepted.<br><br>**Important:** Stating 'data available on request from the author' is not sufficient. If your data are only available upon request, select 'No' for the first question and explain your exceptional situation in the text box.<br><br>Do the authors confirm that all data underlying the findings described in their manuscript are fully available without restriction?	
**Describe where the data may be found in full sentences. If you are copying our sample text, replace any instances of XXX with the appropriate details.**<br><br>• If the data are **held or will be held in a public repository**, include URLs, accession numbers or DOIs. If this information will only be available after acceptance, indicate this by ticking the box below. For example: *All XXX files are available from the XXX database (accession number(s) XXX, XXX.*).<br>• If the data are all contained **within the manuscript and/or Supporting Information files**, enter the following: *All relevant data are within the manuscript and its Supporting Information files.*<br>• If neither of these applies but you are able to provide **details of access elsewhere**, with or without limitations, please do so. For example:<br><br>*Data cannot be shared publicly because of [XXX]. Data are available from the XXX Institutional Data Access / Ethics Committee (contact via XXX) for researchers who meet the criteria for access to confidential data.*<br><br>*The data underlying the results presented in the study are available from (include the name of the third party*	All data files are available from the World Bank database and IMF database.

and contact information or URL).<br>• This text is appropriate if the data are owned by a third party and authors do not have permission to share the data.<br><br>* typeset	
Additional data availability information:	Tick here if the URLs/accession numbers/DOIs will be available only after acceptance of the manuscript for publication so that we can ensure their inclusion before publication.

Powered by Editorial Manager® and ProduXion Manager® from Aries Systems Corporation

Click here to access/download;Manuscript;Manuscript+Anonymous+Topolo

Topological Characteristics of International Business Cycle Synchronization: A Network Analysis of the BRI Economies

ABSTRACT: With the support of social network analysis (SNA), this paper focuses on the topological characteristics of the business cycle synchronization network (BCSN) from overall and individual aspect, which are measured by quasi-correlation coefficient between Belt and Road Initiative (BRI) economies from 2000 to 2019. The research res[illegible]are as follows: First, although there is an overall fluctuation decline in the density and efficiency of BCSN, the BRI strengthen the business cycle synchr2onization relationship; Second, the BCSN presents a topological feature of "small world" which its Topological evolution finally appears to be self-stable; Third, the four subgroups of BCSN show a distribution characteristic of large dispersion and small agglomeration, and BSCN is finally dominated by the two-way overflow groups and broker groups; Fourth, the influence of a country in BCSN don't fully match its intermediary role, individual characteristics between countries and regions are different, and the level of individual characteristics in regions among East Asia, Central Asia and Central and Eastern Europe are relatively higher; Fifth, China's node strength, centrality and coreness have all increased, and China finally occupy the core position of BCSN, but its role as a hub still needs to be further strengthened

Keywords: Belt and Road Initiative (BRI); Business Cycle Synchronization Network (BCSN); Quasi-correlation Coefficient; Social Network Analysis (SNA); Topological Characteristics.

JEL Classification: E3 E32 N10

1. Introduction

The project of Belt and Road Initiative (BRI) was initiated by the Chinese president "Xi Jinping" in 2013, and it was completely introduced during the visit of

Kazakhstan and Indonesia. The creation of novel economic linkages was initiated by developing network of trade, investment, institution, infrastructure and culture among Asia, Europe, Africa, and Middle East (Anwar et al. 2021). The project of BRI comprises of over 50 different economies, which cover 80% of globe population and its the estimated cost is over $21.1 trillion US dollars (Klinger 2019). In 2019, Chinese President Xi Jinping emphasized that, "China will strengthen macro policy coordination with major economies in the world, and jointly promote a strong, sustainable, balanced and inclusive global economy growth".The project of BRI aims to build a community of shared future for mankind, and strength the economic cooperation among partner economies, and now it's moving towards a stage of high-quality construction. The economic, trade, investment, financial and industrial cooperation among countries along the route have been further deepened. At the same time, the ever-expanding circle of friends has promoted political cooperation, policy integration, and systems along the route.

Since the BRI was put forward, more and more countries, regions, organizations, and enterprises had actively responded and deeply participated in. In the context of the changing world as well as new difficulties and obstacles faced by globalization, the BRI has opened up a new cooperation model, activated various cooperation mechanisms, created many cooperation records, promoted regional economic development, and contributed to the globalization. At present, this project is being understood and supported by more and more countries. The economic interaction between China and countries along the route has drawn much attention from the international community, which provides new economic development growth space for countries along BRI route (Huang and Yao 2018). With the expansion of the BRI, on the one hand, the cooperative relationship between different countries in economy, trade, investment, finance and industry has further deepened. On the other hand, there is a large difference between countries along the route in the political, cultural, and economic aspects.

With the continuous acceleration of the international division of labor led by global value chains, the business cycle synchronization development of the business

cycle of different countries leads to a certain dynamic correlation relationship. At the same time, the periodicity and relevance of economic output fluctuations in countries involving in BRI have become more and more significant, and a multi-threaded network spatial relationship also appears. The real economic system has essential characteristics of social networks, it is full of scattered interactions, and the overall economic phenomenon is caused by the interaction of many scattered heterogeneous entities which present multiple relationships such as division of labor, cooperation, trade and so on (Arthur et al. 1997). In fact, the international economy is a complex and dynamic economic system, it contains many complicated economic links which formed by the topological structure. Thus, the SNA is considered as a promising tool for empirically investigating the structures and evolution of international business network (Kurt and Kurt 2020). Furthermore, it is widely used in economic fields such as international trade (Fagiolo 2010), international investment (Bolívar et al. 2019), finance (Chuluun 2017), macroeconomic fluctuations (Lee et al. 2011), industrial economics (Ashton 2008), regional economics (Sigler and Martinus 2017) and so on.

However, the traditional statistic analysis method cannot reveal the complex linkages of business cycles synchronization, so it needs some other methods. Fortunately, social network analysis (SNA) provides a method support for revealing the characteristics of business cycle synchronization among countries, and it has become an emerging research direction (Papadimitriou et al. 2014; Kalyagin et al. 2014; Gomez et al. 2017). This topological approach can bring new interpretation into the evolution of business cycle synchronization relationship, while also allows to reexamine the Topological features of BCSN (Sebestye and Iloskics 2020). Base on the concept of graph theory and social networks, countries can be regarded as nodes, the business cycle synchronization relationship of each country can be regarded as the connecting edges of nodes, and BCSN is formed by the accumulation of many nodes and their connecting edges (Papadimitriou et al. 2014).

Based on the perspective of SNA and existing literature, we found that some related studies have begun to re-examined the business cycle synchronization relationship between countries, and further constructed BCSN for empirical research

(Caraiani 2013; Leiva-Leon 2014; Xi et al. 2014; Sebestyén and Iloskics 2020; Antonakakis et al. 2015). Those studies provided technical support for this paper, but lack of fouces on the countries involving in the BRI. Although there were few studies analyzed the business cycle synchronization between China and other BRI economics (Huang and Yao. 2018; Cui et al. 2020), and investigated the Topological characteristics of BCSN in China and ASEAN region (Du et al. 2020).

At present, several questions about BCSN of the BRI still remain. To be specific, first, what are the temporal and spatial evolution of Topological characteristics of BSCN along the BRI economics? Second, What position important counties occupied in BSCN? Particularly, What are the temporal and spatial changes of China's status in the network? Third, What are the different characteristics of network structure before and after the implementation of BRI ? Finally, as a part of China' s international initiative, under the aims of building a community of shared future for mankind, what are the policy implications for optimizing BSCN structure and enhancing business cycle synchronization relationship between the BRI economics ?

In order to answer these questions, this paper uses quasi-correlation method to measure the level of business cycle synchronization for 53 BRI sample countries in 2000-2019, construct BCSN with the help of SNA base on the business cycle synchronization matrix , and then systematical analysis the structure characteristics of BCSN, in order to make up for the shortage of the existing studies. This article will show a new research perspective for discussing the business cycle synchronization in BRI ~~economics~~ and provide useful policy inspiration for optimizing BCSN structure and enhancing business cycle synchronization in the BRI economies.

Comparing to the previous literature, this paper has three contributions. First, this paper uses quasi-correlation method to systematically examine the business cycle synchronization between the countries (including China) along the ~~route~~, revealing the characteristic facts of the business cycle synchronization relationship. This paper is different from the existing studies (Huang and Yao 2018; Cui et al. 2020), which was only analysis the one-dimension relationship of business cycle synchronization

between China and other countries along the route, and didn’t further reveal the multilateral relationships between other countries. Second, This paper chooses 53 sample countries along the route, and can describe the whole relationship of the business cycle one-dimension in BRI. It is different from Du et al.(2020), which only focused on China and ASEAN, and it is still unable to fully reveal the whole Topological characteristics along the route. Third, this paper will not only analysis the Topological characteristics of BCSN from both overall and individual aspects, but also compare the Topological characteristics between 2000-2013 and 2014-2019, so as to reflect the impact of the BRI on BCSN. Through the comparison of different Topological characteristics of BCSN, and discussing in combination with the real situations, this paper may offer more sufficient and beneficial policy proposals.

The rest of this paper is arranged as follows. Section two shows a literature review related to this topic. Section three presents the methodology explanation and data description. Section four introduces the overall characteristics of BCSN Structure. Section five illustrates the individual characteristics of BCSN Structure.And it is followed by the final section of conclusions and policy implications.

2. Literature Review

International business cycle can be described by simultaneous expansions or recessions of economies, it is also called business cycle synchronization in the literature (Arenas et al. 2008). There are lots of both theoretical and empirical literature in this area, and most of them focused on the measurement and determinants of business cycle synchronization between economies (Frankel and Rose 1998; Rose and Engel 2002; Imbs 2004; Cerqueira and Martins 2009; Karadimitropoulou 2018). Although the above-mentioned existing researches has made great progress and provides solid theoretical and methodological support for this article. It is worth noting that those studies only reflect the business cycle synchronization between one country and other countries, and cannot fully reveal the sophisticated business cycle synchronization relationships between countries. In fact, the international economy is a complex and dynamic economic system. Apparently, uncovering the interaction

structure among economies is the key to understanding the mechanism of business cycle synchronization (Arenas et al. 2008). The most useful way of analyzing network structure of complex synchronization system is to characterize the linkages between nodes through correlation coefficients (Xi et al. 2014).

With the continuous development of social network theories and methods, some studies have analyzed the linkages of business cycle synchronization between countries by means of SNA. Arenas et al. (2008) reported the advances in the comprehension of synchronization phenomena when elements were constrained to interact in a complex network topology, and then reviewed several applications of synchronization in complex networks of economy. Caraiani (2013) found that compared with the Granger causality method, using correlation coefficients to construct a directed business cycle synchronization network can reflect the relative influence of countries in the world economy more reasonably , and further empirical findings showed that the United States finally occupies the core position of the business cycle synchronization network of G7 and OECD. Papadimitriou et al. (2014) used Pearson correlation coefficient and minimum control set to make an empirical study on the structure of the business cycle synchronization network of 22 EU sample member states, and found that after the adoption of the common currency euro, the output of member states had a higher correlation, and the density of the business cycle synchronization network was increasing. Leiva-Leon (2014) established the markov transformation network through the markov zone transformation framework, and found that the American inter-state business synchronization network eventually present a central edge structure of both high and low coordination degree. Xi et al. (2014) measured the internal business cycle synchronization relationship of G7 based on the paired maximum entropy model, and found that the network presented a clustering hierarchy and accounted for 45% of interactive overall relationship structure in G7. Antonakakis et al. (2015) believed that the Sign Concordance Index (SCI) is more suitable than Pearson correlation coefficient to capture the cross-temporal business cycles synchronization between countries.Based on the threshold minimum dominate set (T-MDS) method, the complex network analysis of

business cycle synchronization relationship among 27 countries was carried out. Gomez et al. (2017) used correlation coefficient and minimum spanning tree to construct the EU business cycle synchronization network, analyzed the business synchronization relationship and accessibility among member states, and found that there was no obvious core edge structure inside the network. Sebestyén and Iloskics (2020) employed the Granger causality relationship between national outputs to construct the economic shock contagion network, it is found that the international economic contagion network has a relative long path length and stronger transmission, and the distribution of correspondence tends to be asymmetric.

The research on the BSCN of BRI economies appears to be relatively scarce. Huang and Yao (2018) used CM synchronization index to measure the business cycle synchronization between China and BRI economics without analysing the synchronization relationships between other BRI economics. Cui et al. (2020) used dynamic correlation coefficient to calculate and found that China and Southeast and Central Asian countries, as well as Mongolia, Nepal, Pakistan, Sri Lanka and other countries have a high business cycle synchronization . Du et al. (2020) found that the BRI strengthened the business cycle synchronization relationship between China and ASEAN countries, and the network density and clustering coefficient also increased to some extent.

3. Research Methods and Data Description

3.1 Measurement of Business Cycle Synchronization

In the empirical study, the changing trend of business cycle generated by different method and HP filtering method is basically consistent, and both of these two methods can directly reflect the evolution characteristics of the actual business cycle. Therefore, in order to avoid the measurement errors caused by filtering selection and artificial setting of rolling time window in HP method, as well as the non-dynamic measurement problems in the non-conditional Pearson correlation coefficient method in the same period, which is consistent with Duval et al. (2016), this paper uses quasi-correlation method to measure the business cycle

synchronization of the BRI. The advantage of this method is the traditional correlation coefficient ranging from -1 to 1 not being restricted, it is needless to consider the window period and the selection of filtering method, which can be used for dynamic calculation analysis, and thus is widely used (Abiad et al. 2013; Yao and Tang 2020). The calculation formula of this method is as follows:

$$S_{ijt} = \frac{(y_{it} - \bar{y}_i)(y_{jt} - \bar{y}_j)}{\theta_i \theta_j} \tag{1}$$

Where y_{it} and y_{jt} is the real GDP growth rate of country i and country j in t period, and $\bar{y}_i$ and $\bar{y}_j$ is the mean of real GDP growth rate, θ_i and θ_j is the standard deviation of real GDP growth rate of the two countries, and the real GDP growth rate is measured by the logarithmic difference method. Equation (1) could measure the inter-temporal business cycle synchronization between the two economies in the BRI, and finally form a symmetric quasi-correlation coefficient matrix.

3.2 Social Network Analysis Methods

3.2.1 BCSN Construction

With the continuous advancement of economic globalization and international division of labor, the correlation trend of output periodicity and economic scale among countries presents a certain nonlinear and multi-threaded complex relationship, and then forms an interactive BCSN in space. Therefore, from the perspective of complex system and topological structure, the international business cycle synchronization system is an extremely complex network, rather than a simple collection composed of multiple economies, meanwhile the internal correlation between them should also be considered. In general, a social network is a collection of nodes (social actors) and edges between nodes (relationships between actors) (Scott 2014). Furthermore, when directional features are considered, social networks can be divided into directed networks and undirected networks; When considering the weight characteristics, social networks can be divided into weighted networks and unweighted networks.

According to social network theory and graph theory, BRI economies along the

BRI are regarded as nodes in this paper, and the quasi-correlation coefficients between the actual economic scales of economics are regarded as the connecting edges between nodes. Consistent with Antonakakis et al. (2015), a network (or Graph) is a set of nodes (*N*) linked by edges (*E*), and the mathematical terms could be expressed as: $G=\{N, E\}$, where $V=\{n_1,n_2,...,n_n\}$ represents the node set composed of the BRI economies, $E=\{e_1,e_2,...,e_n\}$ represents the edge set constituted by the quasi-correlation coefficients of economic size between BRI economies.

Considering the symmetry characteristics of the quasi correlation coefficient matrix, when the connecting edges between nodes are quasi correlation coefficients, a weighted undirected BCSN can be constructed. In addition, in the pseudo-correlation coefficient matrix, if the scale value is greater than the mean of the matrix, the value is set as 1, which indicates an actual effective business cycle synchronization relationship edge connection; otherwise, the value is set as 0, which indicates an actual invalid relationship edge connection, so as to be constructed the powerless and undirected. In the matrix data of the above two types of networks, each edge in *E* has a corresponding pair of nodes, and the value of main diagonal of the matrix is zero.

3.2.2 Analysis of the Overall Characteristics of Network Structure

In this paper, indicators such as network density, network efficiency, clustering coefficient, average path length and condensed subgroup are selected to analyze the overall characteristics of BCSN Topological evolution of the BRI.

Network density (DS) describes the degree of business cycle synchronization between the BRI economies, which can be calculated by the ratio of the actual number of relationships to the theoretical maximum number of relationships in the unweighted and undirected network. A more dense network would appear frequent and inseparable shock transmission between the BRI economics, and the shocks originating in other economies usually have high probability of transmitting local turbulence. Network efficiency (NE) reflects the accessibility of economic fluctuation correlation among the BRI economies, and is usually expressed as the reciprocal of the path length between all nodes.

Clustering coefficient (CC) reflects the overall degree of interconnection among

all economies in the network and their immediate neighbors, so as to describe the degree of clustering among some nodes (Fagiolo 2007). Assuming that a node has k edges, the maximum possible number of edges between the nodes (k) connected by these k edges is $k(k-1)/2$, and clustering coefficient can be obtained by dividing the actual number of edges by the maximum possible number of edges. Average path length (APL) represents the mean of the number of connected edges that the shortest path between all potentially connected nodes in the network passes through. The minimum number of edges connecting any two nodes in the network is defined as the path length of these two nodes, and the average of the path lengths of all node pairs in the network is the average path length. It reflects the shocks of transmission efficiency of the business cycle synchronization relationship between nodes. If the network has a large clustering coefficient and a small average path length, it means that the network has small-world characteristics (Watts and Strogatz 1988).

The Quadratic Assignment Procedure (QAP) correlation analysis is a non-parametric test method for calculating the correlations between matrices of different variables based on random matrix permutation (Krackhardt 1988). By observing the changes in matrix correlation at different times, it can effectively reflect the dynamic evolution trend of network structures.

The cohesive subgroups reflect the composition of group subgroups and the tightness between nodes in the network, which is correspondent to subgroup density matrix reflecting the tightness of the business cycle synchronization correlation between each subgroup and its external subgroup. According to the block model theory, network roles can be generally divided into four categories: main beneficiary sector, net spillover sector, broker sector and two-way spillover sector (White et al. 1976). The block model is an important method to explore the network cohesion in social network analysis, it’s commonly used to detect the convergence of the CONCOR algorithm, which is mainly based on the connection between the nodes to identify the network location of the nodes (Snyder and Kick 1979).

3.2.3 Analysis of Individual Characteristics of Network Structure

In this paper, indicators such as node strength, eigenvector centrality,

betweenness centrality and node coreness were selected to analyze the individual characteristics of of BCSN Topological evolution of the BRI. In general, node strength and node coreness could explain absolute influence and status of a country in the network, eigenvector centrality could describe node relative influence, betweenness centrality usually explain the hub position of the node in the network.

Node strength (NS) is the extension of node degree in the unweighted network, which takes into account the number of nearby neighbors of nodes and the weight of connected edges, and is calculated by the sum of the actual effective connected edges between nodes in the unweighted and undirected network and all other nodes, so as to reflect the importance and influence of a node in the cooperative BCSN.

Eigenvector centrality (EC) reflects the relative influence of nodes in the network under different types of network structures and represents the weighted average sum of all direct and undirected connections of each node, that is, its size is affected by the centrality of neighboring nodes (Bonacich 2007).

Betwennness centrality (BC) measures the controlling ability of a node over the whole network, and measured by the proportion of the number of nodes that need to pass through the shortest path among all nodes in the network.

Node coreness (NC) reflects the core status of a node in the network, and reveals the special structure of the business cycle synchronization between the core and peripheral nodes in the network through core edge analysis.

3.3 Data Description and Source

Real GDP data is expressed as GDP in US dollars in 2010, and the data comes from the World Development Indicators (WDI) database in the World Bank. To the choice of sample, we refer to the standards from China's BRI website (*https://www.yidaiyilu.gov.cn/*). Comprehensively considering the availability and consistency of data, 53 sample BRI economics from 2000 to 2019 are selected as the research objects in this paper. More details of sample countries are available in Appendix (Table A1).

4. BCSN Structure: Analysis of Overall Characteristics

4.1 Analysis of Network Topological Form

As can be seen from Table 1, the network density and network efficiency of the BCSN from 2000 to 2019 have obvious stage characteristics, showing a fluctuating downward trend with rise and fall coexist on the whole, indicating that the business cycle synchronization relationship between nodes is still in a state of relatively weak connection, and the accessibility of the business synchronization correlation is general. Specifically, from 2000 to 2008, network density increased as a whole and was higher than the average in the sample period, while network efficiency increased firstly and then decreased. From 2008 to 2012, the overall network density and efficiency decreased, and gradually fell below the mean value. The network density and efficiency had an increasing overall fluctuation from 2014 to 2019.

It is shown that the real and effective synchronization relationship between countriesinvolving in BRI from 2000 to 2012 was obviously adversely affected by the 2008 global financial crisis, and the accessibility of economic fluctuation and risk correlation was also rapidly declining, which also indicated that countries adopted relatively active anti-business cycle prevention policies to resist the impact of the financial crisis. After the BRI was put forward in 2013, the closeness of BCSN has been strengthened, and the accessibility of corresponding economic links has also been improved, indicating that BRI aimed at building a mutually beneficial and win-win "community of shared future" had a positive impact on enhancing economic and trade cooperation and links among the BRI economics.

Table 1 Statistical results of overall characteristics.

Indicator	2000	2002	2004	2006	2008	2010	2012	2014	2016	2018	2019	Mean
DS	0.470	0.495	0.477	0.462	0.504	0.495	0.395	0.388	0.382	0.532	0.427	0.455
NE	0.493	0.495	0.535	0.573	0.495	0.495	0.429	0.441	0.434	0.752	0.462	0.511
CC	0.964	0.999	0.889	0.922	1.000	1.000	0.927	0.913	0.892	0.942	0.959	0.941
WCC	0.985	0.998	0.820	0.861	1.000	1.000	0.929	0.893	0.851	0.916	0.968	0.925
APL	1.057	1.001	1.290	1.406	1.000	1.000	1.142	1.227	1.253	1.560	1.150	1.230

Note: Calculated by the authors from UCINET6.

It is worth noting that in 2019, network density and efficiency saw a significant decline, which was mainly caused by the unilateral trade protectionism and continuous trade frictions among western countries led by the US in recent years, which worsened the previous positive global economic and trade environment.

Furthermore, the clustering coefficient decreased on the whole and fluctuated around 0.941, while the corresponding weighted clustering coefficient (WCC) was around 0.925. Both of them reached their peaks during 2008-2010, indicating that the synchronization relations among some nodes in the network had obvious clustering characteristics. Meanwhile, the impact of the financial crisis made the clustering of node relations more obvious. The average path length increased slightly on the whole and fluctuated around 1.230, proving that the path required for the transmission of the synchronization relationship is relatively short, that is, the economic output and fluctuations of any country in the network only need to be transmitted once to reach other countries. In particular, both network clustering coefficient and average path have increased from 2014 to 2019, showing that the business cycle synchronization relationship of countries under the BRI has been strengthened, and the correlation between economic output and fluctuations have become more direct. Therefore, the BCSN of the BRI economics always show a large clustering coefficient and a small average path length, which has the typical "small world" Topological characteristics.

4.2 Analysis of Network Structure Evolution

From Table 2, in 2000-2013 business cycle synchronization matrix along the route show obvious weak related or unrelated characteristics, and its network structure evolution does not have the typical self-stable characteristic. However, from 2014 to 2019, the positive correlation of synchronization matrix was gradually strengthened, the characteristics of gradualism structure evolution began to highlight at this time.

Specifically, from 2000 to 2012, the correlation coefficient between the business cycle synchronization matrix is basically near zero value, and most of them fail to pass the significance test.Taking the year 2000 as an example, the correlation between

the synchronization matrix of 2000 and 2002 is only -0.014, and the correlation coefficient between 2000 and subsequent years is only between -0025 and 0.065, both of which fail to pass the significance test, indicating that the overall network structure does not evolve step by step, and the evolution of network structure in the previous period will not significantly affect the change in the later period. Although the correlation of the synchronization matrix in each year from 2014 to 2019 is still low, it has gradually become significantly positive. The correlation coefficient is 0.097 between 2016 and 2018 ,and that is 0.236 between 2018 and 2019. Meanwhile, the correlation between 2018 and 2019 reaches 0.298. It is indicated that with the proposal and practice of BRI, the business cycle synchronization of the BRI economics began to show a dynamic and increasing positive correlation, and there was a significant mutual influence on the evolution of network structure in the early and late period.

Table 2 QAP correlation result.

QAP	2000	2002	2004	2006	2008	2010	2012	2014	2016	2018	2019
2000	1.000										
2002	-0.014	1.000									
2004	0.065	-0.004	1.000								
2006	0.032	0.007	-0.048	1.000							
2008	0.005	-0.018	-0.017	-0.012	1.000						
2010	0.032	-0.018	-0.017	-0.018	-0.010	1.000					
2012	0.035	0.033*	0.076	0.162**	-0.004	0.041*	1.000				
2014	0.052	0.06**	0.053	0.184**	-0.013	0.005	0.104**	1.000			
2016	0.027	0.012	-0.043	0.081	-0.016	-0.010	-0.144**	0.111*	1.000		
2018	-0.025	-0.012	-0.039	-0.007	0.004	0.033	-0.021	0.017	0.097***	1.000	
2019	0.001	-0.024	-0.002	0.013	-0.025	0.022	-0.032	0.054	0.236***	0.298***	1.000

Note: Calculated by the authors from UCINET6.

4.3 Structure Analysis of Network Subgroup

In order to better reveal the subgroup structure changes of BCSN of the BRI economics before and after the proposal of the BRI, this paper divides the synchronization matrix over the past years into 2000-2013 mean matrix and 2014-2019 mean matrix. Finally, the table of density matrix change and group relationship between 2014 and 2019 are obtained , as well as the subgroup structure in two periods (Figure 1 and Figure 2). In those two figures, the circle, square, triangle, and box represents the first, second, third and fourth group respectively.

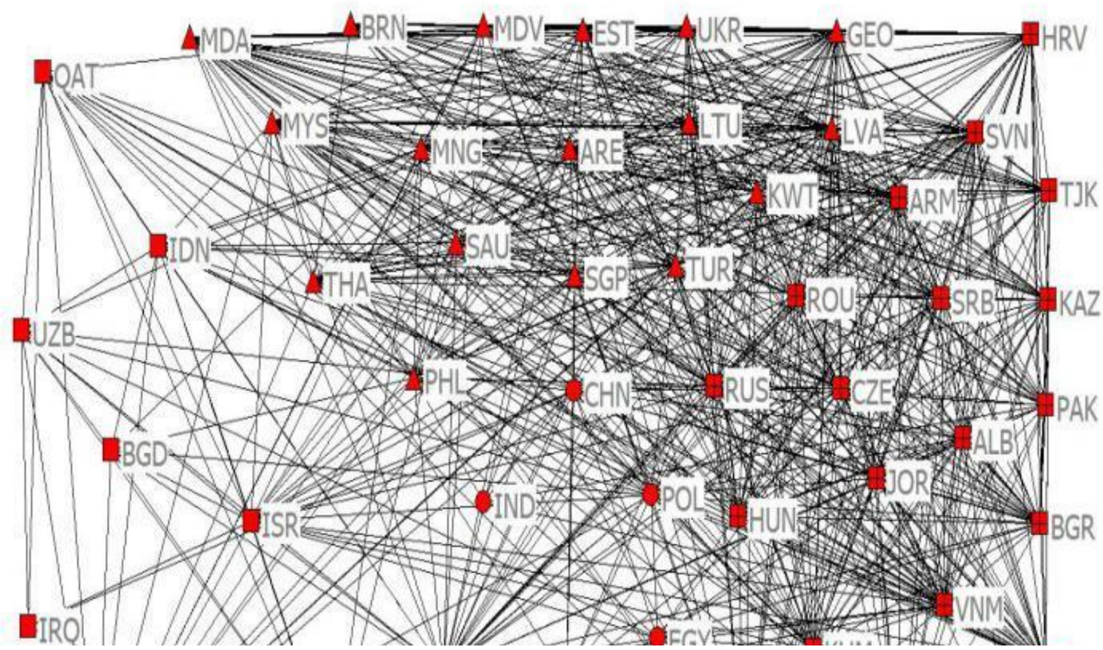

Fig. 1. BCSN subgroup structure in 2000-2013(Left) and 2014-2019(Right).

Source: ~~Drew~~ by the authors from UCINET6 software's NetDraw function.

It can be seen that BCSN structure of the BRI economics is composed of four major subgroups, which show obvious co-existing characteristics of large dispersion and small aggregation in space. There are great differences among different subgroups in terms of the synchronization degree and its changes, which the one for inner

subgroups is much greater than that of external subgroups. To be specific, compared with 2000-2013, the synchronization degree of internal nodes in subgroup1 is relative high and has the largest increase during 2014-2019.

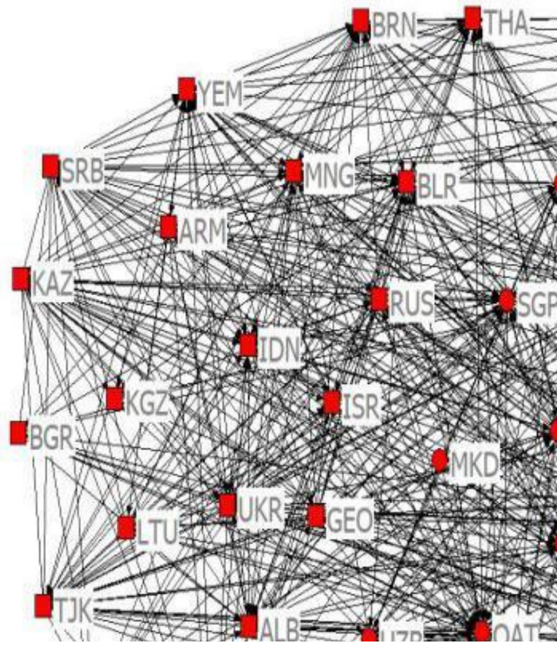

Fig. 2. BCSN subgroup structure in 2000-2013(Left) and 2014-2019(Right).

Source: Drew by the authors from UCINET6 software's NetDraw function.

Secondly, the synchronization degree with subgroup 3 is enhanced, while the one with subgroup 2 and 4 is significantly decreased. The number of nodes in the subgroup 1 has increased, mainly including countries such as China, Singapore and Turkey as well as two isolated nodes (Nepal and Yemen). The number of internal received relations of the subgroup 1 is 222, the number of external sent relations is 205, the corresponding expected relation ratio is 0.308, and the actual relation ratio is 0.520, indicating that the subgroup 1 is a two-way spillover group.

Subgroup 2 mainly contains India, Israel, Russia and other countries, and the number of its internal nodes has increased significantly. In addition to the decline of

synchronization degree of internal connection, the synchronization degree of external connection has increased significantly. The number of internal received and external sent relations of subgroup 2 is 290 and 244 respectively, and the expected relation ratio and actual relation ration is 0.365 and 0.543 respectively, which has typical broker group characteristics and plays a pivotal role in the process of business cycle synchronization development.

The number of internal nodes of subgroup 3 decreased significantly, and a relatively isolated subgroup containing only Indonesia and Malaysia was finally formed. At this time,the internal synchronization degree and the one of the relationship between the subgroup 2 and 3 increased, but the synchronization degree with subgroup 4 decreased. The number of relationships between internal received and external sent of the subgroup 3 is 2 and 17, respectively, and the expected relation ratio and actual relation ratio is 0.019 and 0.105, respectively, showing obvious outward spillover characteristics and belonging to the net spillover group. Although the internal business synchronization relationship of this group is very close, the number of its internal business synchronization relationship is relatively small due to the sharp decrease of the number of nodes to two, while the number of its external business synchronization relationship is significantly much more.

The number of internal nodes in subgroup 4 has decreased and the internal tightness degree of connection have decreased, mainly including countries like Vietnam, the Philippines, Pakistan, Poland and Romania. The number of received and sent relations of the fourth group is 142 and 60, and the expected relation ratio and actual relation ratio is 0.250 and 0.703, respectively. It is a typical beneficiary in the BCSN and belongs to the beneficiary group. From the perspective of network structure evolution, the network structure was dominated by subgroup 3 and subgroup 4 from 2000 to 2003, while subgroup 1 (two-way spillovers) and subgroup 2 (brokers) constituted the main part of the network from 2014 to 2019, which indicates that the BRI has strengthened the business cycle synchronization relationship of the BRI economics as well as a radiation-related structure is formed in space with China, Singapore, India, Saudi Arabia, Turkey, Russia and Kazakhstan acting as the main

nodes.

Table 3 Density matrix variation and group relations.

	Changes of Density Matrix				Subgroup Links Division in 2014-2019							
	S1	S2	S3	S4	S1	S2	S3	S4	Number of Nodes	Expected Relation Ratio	Actual Relation Ratio	Role
S1	0.816 (0.399)	(-0.201)	(0.088)	(-0.343)	222	191	11	3	17	0.308	0.520	Two-way Spillover group
S2	0.562	0.763 (-0.094)	(-0.202)	(0.105)	191	290	1	52	20	0.365	0.543	Broker group
S3	0.324	0.025	1.000 (0.100)	(-0.455)	11	1	2	5	2	0.019	0.105	Net Spillover group
S4	0.013	0.186	0.179	0.780 (-0.083)	3	52	5	142	14	0.250	0.703	Primary Benefit group

Note: 1.Calculated by the authors from UCINET6 software's CONCOR algorithm. The S represent subgroup. The density matrix is symmetric, and the values in brackets are the changes of density values from 2014 to 2019 compared with 2000-2013. 2.Expected relation ratio = (the number of nodes in the group minus one) / (the number of all nodes minus one), and actual relation ratio = the number of internal contacts in the subgroup/the total number of external links issued by the subgroup.

5. BCSN Structure: Analysis of Individual Characteristics

In order to better reveal the evolution of individual characteristics of BCSN structure before and after the implement of BRI, this section will mainly show the individual characteristic values and their changes and corresponding rankings in key

node countries and different regions from 2014 to 2019 (Table 4), and analyze the results and rankings of China's characteristic values in major years (Table 5).

5.1 Analysis of Node Strength

Table 4 shows that, on the whole, node strength of important node countries and regions from 2014 to 2019 presents typical disequilibrium characteristics, and decreases to varying degrees compared with 2000-2013. It is indicated that node countries have significant differences in their role of business synchronization within their regions, and due to the adverse impact of the 2008 financial crisis, the development of business cycle synchronization along the route is facing greater uncertainty. Furthermore, South Asia ranked the last in node strength. The node strength of India and Pakistan, the node countries in the region, was less than zero (-5.495 and -3.761), and showed a significant decline.This indicates that South Asia is in the process of counter-economic cycle development, and the node countries have not played their due positive synchronization coordination role, which is not conducive to the positive synchronization development of the overall economy along the route. Southeast Asia, with a big decline, ranked fifth in node intensity, while the node countries in the region ranked 14th and 15th in node intensity, respectively, which indicated significant differences in node intensity among countries in the region, Singapore and Thailand are leading the business synchronization development relationship in the region and abroad.

Central and Eastern Europe (CEE) was the fourth largest region in terms of node strength, but saw the biggest drop (-23.932), while Russia and Ukraine ranked 4th and 11th within the region in terms of node strength,which indicated that there was a significant difference in the strength of nodes within CEE, and relying on the original political and economic cooperation, Russia led the synchronization development of central and Eastern Europe and its external business cycle.

Central Asia ranked third, while Kazakhstan and Tajikistan ranked second and 13th, respectively in terms of node strength, indicating that Kazakhstan played an important role in the business cycle synchronization development in Central Asia.

Although West Asia and North Africa ranked second in terms of node strength

while Saudi Arabia and Egypt ranked relatively low, indicating significant national differences in node strength within the region, but the role of Saudi Arabia and Egypt was not obvious.

Finally, East Asia, which only includes China and Mongolia, had the highest corresponding node strength (8.341), with a relative decline of -8.446. China's node strength was 10.127 and ranked the first, while Mongolia ranked the tenth, which indicated that East Asia played an important leading role in the process of business cycle synchronization in the BRI economies.

It can be seen that China, Singapore, Kazakhstan, Russia and other countries play an important nodal role, leading business cycle synchronization relationship between the regional and external business cycles, while the role of Saudi Arabia and Egypt is not obvious.

Further combined with Table 5, it can be seen that in the sample period, the node strength of China showed an overall fluctuating growth trend of rising first and then falling, and rose to the first place in 2019. There were significant differences in node strength around 2008. It is suggested that as the initiator of the BRI, China has gradually integrated into and finally played a dominant role in the business synchronization relationship among the countries involving in the BRI. It is in the track of positive business cycle development with most of the BRI economics and plays an important role in promoting the business cycle synchronization development among the BRI economics.

To be specific, from 2000 to 2006, China's node strength rose rapidly from 0 to 75.819, and its ranking also rose rapidly to the fourth place, which indicates that during this stage, after China's entering the WTO, it was rapidly integrating into the business cycle synchronization development of countries involving in the BRI, and gradually occupying the core position of the synchronization relationship network. From 2008 to 2012, China's node strength dropped sharply to between 1.880 and 8.462, ranking was between 13 and 24.

At this time, due to the impact of the financial crisis, the business cycle synchronization relationship between China and the countries involving in the BRI is

facing relatively greater uncertainty.

Table 4 Statistics of individual characteristics of important countries in 2014-2019.

Country/ Region	Node Strength			Eigenvectors Centrality			Betweenness Centrality			Node Coreness		
	SG	Changes	Rank	EC	Changes	Rank	BC	Changes	Rank	NC	Changes	Rank
China	10.127	-7.750	1	0.186	0.038	3	3.280	-3.710	23	0.188	0.070	5
Mongolia	6.555	-9.142	10	0.134	0.058	18	4.784	3.208	19	0.177	0.064	14
Singapore	5.765	-16.644	14	0.182	0.054	10	1.256	-0.021	40	0.182	0.044	7
Thailand	5.552	-12.535	15	0.121	0.012	22	4.037	2.676	21	0.182	0.044	7
India	-5.495	-10.266	51	0.061	-0.036	43	4.965	4.095	18	0.068	0.038	43
Pakistan	-3.761	-20.237	49	0.037	-0.041	48	0.260	-1.572	48	0.063	-0.055	46
Saudi Arabia	2.530	-13.996	24	0.122	-0.001	21	1.757	0.176	32	0.148	0.040	24
Egypt	0.531	-11.361	36	0.069	-0.036	39	2.086	-0.269	29	0.086	0.007	37
Russia	9.081	-20.498	4	0.188	0.036	1	5.446	4.360	16	0.182	-0.010	7
Ukraine	6.533	-19.201	11	0.074	-0.058	36	138.189	136.219	1	0.200	0.037	1
Kazakhstan	9.651	-8.120	2	0.186	0.047	4	5.153	2.171	17	0.200	0.057	1
Tajikistan	6.321	-8.744	13	0.185	0.092	7	44.393	41.606	3	0.177	0.049	14
East Asia	8.341	-8.446	1	0.160	0.048	1	4.032	-0.251	4	0.183	0.067	1
Southeast Asia	1.983	-18.689	5	0.117	0.012	4	2.845	0.727	5	0.133	-0.007	4
South Asia	-2.039	-11.418	6	0.067	-0.021	6	1.765	-1.126	6	0.101	0.031	6
West Asia & North Africa	3.903	-13.635	2	0.128	0.022	3	6.314	3.359	3	0.139	0.024	2
Central & Eastern Europe	2.037	-23.932	4	0.092	-0.032	5	18.908	17.000	1	0.118	-0.047	5
Central Asia	3.821	-5.803	3	0.139	0.041	2	10.642	8.440	2	0.135	0.055	3

Note: Calculated by the authors from UCINET6. The change value was the difference between 2014-2019 and 2000-2013, and the ranking was compiled according to the results of

2014-2019. The regional result is the mean of the sample eigenvalues in the region.

Table 5 Statistics on individual characteristics of China in 2000-2019.

Indicator	2000	2002	2004	2006	2008	2010	2012	2014	2016	2018	2019
SG	-3.265	-0.472	20.704	75.819	1.880	3.413	8.462	10.036	16.093	2.067	11.691
	(41)	(26)	(36)	(4)	(24)	(13)	(21)	(7)	(7)	(2)	(1)
EC	0.000	0.180	0.088	0.184	0.186	0.186	0.182	0.190	0.194	0.170	0.184
	(34)	(28)	(36)	(4)	(1)	(1)	(20)	(7)	(7)	(21)	(1)
BC	0.000	0.000	0.000	43.208	0.000	0.186	0.000	5.931	6.921	0.000	0.160
	(13)	(28)	(27)	(4)	(10)	(1)	(10)	(7)	(7)	(31)	(13)
NC	0.067	0.144	0.075	0.205	0.149	0.149	0.169	0.191	0.198	0.141	0.168
	(46)	(28)	(36)	(4)	(1)	(1)	(20)	(7)	(7)	(23)	(1)

Note: Calculated by the authors from UCINET6. The values in brackets are the rankings.

In addition, the different policies from different countries to deal with financial risks make the business cycle more difficult to synchronize. From 2014 to 2019, China's node strength showed a certain fluctuating growth, and the corresponding ranking finally ranked the top one, which indicated that in the post-crisis era and the China and US trade dispute period, China had gradually played a leading role in the business cycle synchronization relationship among the BRI economics.

5.2 Analysis of Node Centrality

It can be seen from Table 4 that, on the whole, the eigenvector centrality and intermediary centrality of countries with important nodes are relatively average. There are obvious transnational differences within different regions, which indicate that the relative influence of important node countries in the whole BCSN still needs to be improved, and they have not played the role of "hub" and "bridge" effectively.

Further comparison shows that the relative influence and mediating role of some nodal countries, such as China, Russia, Ukraine, Kazakhstan and Tajikistan, do not match, but their overall performance is better than that of other nodal countries.

Specifically, eigenvector centrality of East Asian ranked top one, but its intermediate centrality ranked fourth. China's eigenvector centrality improved, but intermediate centrality decreased significantly, which indicate that East Asia occupies a certain relative influence, but the role of the hub is not outstanding.

Combined with Table 5, it can be found that the relative status and influence of China in the whole network during the sample period have significantly improved, and the performance was better after implementation of BRI, and the performance of corresponding intermediary centrality was average. It is suggested that China's pivotal role in the BCSN still needs to be improved due to the various differences among BRI economies and the influence of external uncertainty.

Although Southeast Asia's overall centrality level has improved, it remains relatively low in the rankings, among which Singapore's relative influence is better than Thailand's, but Thailand's role of hub performs better. In South Asia, the centrality of the two categories declined to varying degrees, ranking the last meanwhile the centrality level of Pakistan and India in the region is similar to its node strength, and it does not have a certain influence and mediating effect on the regional and external business synchronization relationship.Both West Asia and North Africa have improved their centrality level to varying degrees and rank in the median overall, among which the relative influence of nodal countries like Saudi Arabia and Egypt have decreased and these two countries do not play an obvious role as hubs.

There is a significant difference in the level of the two types of centrality in Central and Eastern Europe, and their relative influences are somewhat reduced and far less than the influence of the pivotal role they played. There is also a significant difference in the performance of the node countries in the region. The relative influence of Russia finally ranks the first, but its intermediary role shows that Ukraine occupies the core hub position in the transmission of business synchronization relationships in the regions. Central Asia is deeply landlocked and geographically close to China, ranking second in centrality, in which Kazakhstan has a higher relative influence and Tajikistan plays a more prominent intermediary role.

After comparison, it is found that the relative influence of East and Central Asia

at both ends of the route is prominent, while the intermediate role of Central and Eastern Europe and Central Asia performs better, which provides support and direction for the positive and business cycle synchronization development of the BRI economics.

5.3 Analysis of Node Coreness

Table 4 shows that, on the whole, the coreness level of the BCSN performs a certain feature of "multi-center and wide edge", which is composed of multiple levels of cores, semi-cores and edges. Among them, East Asia, West Asia and North Africa have a higher coreness level and occupy the relative core position of the network, while Central Asia and Southeast Asia are in the intermediate zone between the core and the edge of the network, Central and Eastern Europe and South Asia are in the edge of the network.

Further combined with Table 5, it can be seen that China's coreness level fluctuated from 0.068 to 0.168 during the sample period. The corresponding ranking reached first, which was similar to the eigenvector centrality performance, and performed significantly better in the process of coping with the impact of the financial crisis and trade disputes. It is shown that during the sample period, China experienced WTO accession, financial crisis, economic supply-side Topological reform, the proposal and construction of the BRI, and trade disputes between the US and China and it is accelerating its integration into the BCSN, and eventually occupies the core position of the network.This may be due to China's strong driving force for economic growth and higher level of opening-up policy, as well as its systematic and reliable economic and financial risk prevention policy, and the formation of a good and close relationship of coordinated economic development with the BRI economics.

From the perspective of different regions, although the coreness level of Mongolia is obviously worse than that of China, the gap between the two is small, contributing to the top ranking of East Asia as a whole. Singapore and Thailand achieved synchronized growth in coreness and tied for seventh place, but other countries in the region did not achieve high coreness level, resulting in the overall lagging behind. In South Asia, Pakistan and India are significantly lower in the

coreness level and ranking, and at the edge of the network. The coreness level of West Asia and North Africa has been improved and ranks second, but the core influence of Egypt and Saudi Arabia in the regional and external business synchronization relationship is not prominent. Central and Eastern Europe ranked last in terms of coreness level, with Ukraine significantly superior to Russia. Central Asia has improved its coreness level, and Kazakhstan's core position is obviously better than Tajikistan. Further comparison shows that although Central and Eastern Europe has the lowest coreness level, Ukraine and Kazakhstan share the first place in coreness level from 2014 to 2019. Such obvious differences in internal coreness level also exist in other regions except East Asia and South Asia.

6. Conclusion

Under the support of quasi-correlation coefficient and social network analysis method, this article empirically analyzed the Topological characteristics of BCSN of BRI economies from 2000 to 2019. The main research conclusions are as follows:

Firstly, in the sample period, the density and efficiency of BCSN show a fluctuating downward trend, the synchronization relationship is relatively weak, and the accessibility of the correlation between economic fluctuations and risks is general. After the BRI was put forward, the closeness and accessibility of economic cooperation along the route have been strengthened, but they are still affected by unstable factors such as trade disputes.

Secondly, BCSN always show a large clustering coefficient and a short average path length, which is typical of "small world" structure. From 2000 to 2013, the synchronization matrices of the business cycle showed obvious weak correlated or uncorrelated feature, and the evolution of the network structure did not have the characteristic of the typical step-by-step evolution. However, the positive correlation of the synchronization matrices increased gradually from 2014 to 2019, and the Topological evolution showed certain self-stable characteristics.

Thirdly, BCSN structure consists of four subgroups, and the spatial distribution of them is characterized by the coexistence of large dispersion and small aggregation,

the synchronization degree between different subgroups and its change show a great difference. The synchronization degree inside subgroups was obviously higher than that of the external subgroups. From 2014 to 2019, the network structure was dominated by two-way spillover sectors and broker sectors with a large number of nodes. More important, China belongs to two-way spillover sectors, and plays an important role in BCSN.

Fourthly, the individual characteristics of the BRI economics are obviously different, the core status and influence of a country in the network don't fully match its hub role. In 2008, the financial crisis prompted the business cycle synchronization relationship to face greater uncertainty, resulting in a significant decline in node strength. However, after the implementation of the BRI, the role of China and important nodes, such as Southeast Asia, Central Asia and Central and Eastern Europe, has been enhanced. East Asia occupies a relatively high position in the network, Central and Eastern Europe and Central Asia play the most prominent intermediary role while Southeast Asia, West Asia and North Africa perform relatively normal, and South Asia performs significantly less well.

Fifthly, China's node intensity, eigenvector centrality and coreness achieved a fluctuating growth on the whole, and finally ranked top one, indicating that China was accelerating its integration into BCSN and strengthening the business cycle synchronization relationship with BRI economics. Although China's core position and influence in the network was relatively prominent, its role as an intermediary hub still needed to be further strengthened.

Some policy implications could be obtained from our study. Firstly, as an key advocate of the BRI, China speed up the construction of hardware and software infrastructures for connectivity along BRI economies, actively ~~carry~~ out high-quality international cooperation in various fields and expand the BRI friend group by enhancing political dialogue and cultural communication, flexibly apply systemic economic risk prevention tools to play the role of key nodes in the BCSN. Secondly, China should continue to play the core influence in BCSN and enhance its role as a coordinating hub in the business synchronization development relations along the

route, to formulate a comprehensive multilateral risk early warning mechanism for the BRI economies, to strengthen cooperation of economic risk and epidemic prevention with BRI economics, in order to optimize the structure of BCSN. Thirdly, China should give full play to the role of RMB internationalization in realizing financial integration along the route, accelerate the development of high-quality and diversified international cooperation with countries at key junctions, develop differentiated models of international economic cooperation for different regions and countries.

Acknowledgments

We are grateful to anonymous reviewers for constructive and insightful comments. All remaining errors are the responsibility of the authors alone.

Appendices1. List of 53 samples ~~economics~~ along the BRI

Table.A1 53 samples economics along the BRI

ID	NAME	ISO	Geographic Regions	ID	NAME	ISO	Geographic Regions
1	China, P.R.	CHN	East Asia	28	Iraq	IRQ	West Asia & North Africa
2	Mongolia	MNG	East Asia	29	Azerbaijan	AZE	West Asia & North Africa
3	Russia	RUS	Central & Eastern Europe	30	Georgia	GEO	West Asia & North Africa
4	Singapore	SGP	Southeast Asia	31	Armenia	ARM	West Asia & North Africa
5	Indonesia	IDN	Southeast Asia	32	Poland	POL	Central & Eastern Europe
6	Malaysia	MYS	Southeast Asia	33	Albania	ALB	Central & Eastern Europe
7	Thailand	THA	Southeast Asia	34	Estonia	EST	Central & Eastern Europe
8	Vietnam	VNM	Southeast Asia	35	Lithuania	LTU	Central & Eastern Europe
9	Philippines	PHL	Southeast Asia	36	Slovenia	SVN	Central & Eastern Europe
10	Cambodia	KHM	Southeast Asia	37	Bulgaria	BGR	Central & Eastern Europe
11	Brunei	BRN	Southeast Asia	38	Czech Republic	CZE	Central & Eastern Europe
12	India	IND	South Asia	39	Hungary	HUN	Central & Eastern Europe
13	Pakistan	PAK	South Asia	40	Republic of Macedonia	MKD	Central & Eastern Europe
14	Sri Lanka	LKA	South Asia	41	Serbia	SRB	Central & Eastern Europe
15	Bangladesh	BGD	South Asia	42	Romania	ROU	Central & Eastern Europe

16	Nepal	NPL	South Asia	43	Slovakia	SVK	Central & Eastern Europe
17	Maldives	MDV	South Asia	44	Croatia	HRV	Central & Eastern Europe
18	Bhutan	BTN	South Asia	45	Latvia	LVA	Central & Eastern Europe
19	United Arab Emirates	ARE	West Asia & North Africa	46	Ukraine	UKR	Central & Eastern Europe
20	Kuwait	KWT	West Asia & North Africa	47	Belarus	BLR	Central & Eastern Europe
21	Turkey	TUR	West Asia & North Africa	48	Moldova	MDA	Central & Eastern Europe
22	Qatar	QAT	West Asia & North Africa	49	Kazakhstan	KAZ	Central Asia
23	Saudi Arabia	SAU	West Asia & North Africa	50	Kyrgyzstan	KGZ	Central Asia
24	Israel	ISR	West Asia & North Africa	51	Turkmenistan	TKM	Central Asia
25	Yemen	YEM	West Asia & North Africa	52	Tajikistan	TJK	Central Asia
26	Egypt	EGY	West Asia & North Africa	53	Uzbekistan	UZB	Central Asia
27	Jordan	JOR	West Asia & North Africa				

References

Abiad, A., D. Furceri, S. Kalemli-Ozcan, and A. Pescatori. 2013. “Dancing Together? Spillovers, Common Shocks, and the Role of Financial and Trade Linkages.” Washington: International Monetary Fund,World Economic Outlook 81-111.

Antonakakis, N., P, Gogas, T, Papadimitriou, and G. A. Sarantitis. 2015. “International Business Cycle Synchronization Since the 1870s: Evidence from a Novel Network Approach.” Physica A: Statistical Mechanics and its Applications 447(14):286-296.

Anwar, M. A., S. Nasreen, and A. K. Tiwari. 2021. “Forestation, Renewable Energy and Environmental Quality: Empirical Evidence From Belt and Road Initiative Economies.” Journal of Environmental Management 291(8):112684-112683.

Arenas, A., A. Diaz-Guilera, J. Kurths, Y. Moreno, and C. Zhou. 2008. “Synchronization in Complex Networks.” Physics Reports 469(3):93-153.

Arthur, W. B., Durlauf, S. N. and D. A. Lane. 1997. “The Economy as an Evolving Complex System II.” MA: Addison-Wesley, Advanced Book Program, 65:337-367.

Ashton, W. 2008. “Understanding the Organization of Industrial Ecosystems : A Social Network Approach.” Journal of Industrial Ecology 12(1):34-51.

Bolívar, L. M., C. Casanueva, and I. Castro. 2019. “Global Foreign Direct Investment: A Network Perspective.” International Business Review 28:696712.

Bonacich, P. 2007. “Some Unique Properties of Eigenvector Centrality”. Social Networks 29(4): 555-564.

Caraiani, P. 2013. “Using Complex Networks to Characterize International Business Cycles.” PLOS ONE 8(3):1-13.

Cerqueira, P. A., and R. Martins. 2009. “Measuring the Determinants of Business Cycle Synchronization Using A Panel Approach.” Economics Letters 102(2):106-108.

Chuluun, T. 2017. “Global Portfolio Investment Network and Stock Market Co-movement.” Global Finance Journal 33(5):51-68.

Cui, Q. Y., Y. Zhang, and S. Wang. 2020. “International Macroeconomic Policy Coordination Under the BRI: Mechanism Basis and China’s Role.” Economists (8):49-58.

Du, J. Z., C. Z., Wang, and J. L. Wang. 2020. “An Analysis of Business Cycle Synchronization Between China and ASEAN Countries in the Context of the Belt and Road Initiative.” Journal of Finance and Economics Theory (4):23-31.

Duval, R., N. Li, R. Saraf, and D. Seneviratnea. 2016. “Value-added Trade and Business Cycle Synchronization.” Journal of International Economics 99:251-262.

Fagiolo, G. 2007. “Clustering in Complex Directed Networks”. Physical Review E 76(2):0261071 -2061078.

Fagiolo, G., J. Reyes, and S. Schiavo. 2010. “The Evolution of the World Trade Web: A Weighted-network Analysis.” Journal of Evolutionary Economics 20(4):479-514.

Frankel, J.A., and A. K. Rose. 1998. “The Endogenity of the Optimum Currency Area Criteria.” The Economic Journal 108(449): 1009-1025.

Gomez, D. M., H. J. Ferrari, B. Torgler, and J. O. Guillermo. 2017. “Synchronization and Diversity in Business Cycles: A Network Analysis of the European Union.” Applied Economics 49(10):1-15.

Huang, Z. L., and T. T. Yao. 2018. “Business Cycle Synchronization and its Transmission Mechanism between China and the Countries along the BRI.” Statistical Research 35(9): 40-53. (In Chinese).

Imbs, J. 2004. “Trade, Finance, Specialization, and Synchronization.” Review of Economics and

Statistics 86(3):723-734.

Kalyagin, V. A., P. M. Pardalos, and T. M. Rassias. 2014. “Network Models in Economics and Finance.” Switzerland: Springer International Publishing.

Karadimitropoulou, A. 2018. “Advanced Economies and Emerging Markets: Dissecting the Drivers of Business Cycle Synchronization.” Journal of Economic Dynamics & Control 93(8): 115-130.

Krackhardt, D. 1988. “Predicting with Networks: Non-parametric Multiple Regression Analysis of Dyadic Data.” Social Networks 10(4):359-381.

Lee, K. M., J. S. Yang, G. Kim, J. Lee, K. I. Goh, et al. 2011. “Impact of the Topology of Global Macroeconomic Network on the Spreading of Economic Crises.” PlOS ONE 6(3):e18443.

Leiva-Leon, D. 2014. “A New Approach to Infer Changes in the Synchronization of Business Cycle Phases.” Ottawa: Bank of Canada Working Paper, No. 2014-38.

Kurt, Y. and Kurt, M. 2020. “Social Network Analysis in International Business Research: An Assessment of the Current State of Play and Future Research Directions.” International Business Review 29(2):101633.

Klinger, J. 2019. “Environment, Development, and Security Politics in the Production of Belt and Road Spaces.” Territory Politics Governance 8(5):657-675.

Papadimitriou, T., P. Gogas, and G. A. Sarantitis. 2014. “European Business Cycle Synchronization: A Complex Network Perspective, Network Models in Economics and Finance.” The Rimini Centre for Economic Analysis Working Paper, No.13_44,13:265-275.

Rose, A. K. and C. Engel. 2002. “Currency Unions and International Integration.” Journal of Money, Credit, and Banking 34(4):1067-1089.

Scott, J. P. 2014. “The SAGE Handbook of Social Network Analysis.” London: Sage Publications.

Sebestyén, T., and Z. Iloskics. 2020. “Do Economic Shocks Spread Randomly?: A Topological Study of the Global Contagion Network.” PLOS ONE 15.e0238626:1-22.

Sigler, T. J. and K. Martinus. 2017. “Extending Beyond 'World Cities' in World City Network (WCN) Research: Urban Positionality and Economic Linkages Through the Australia-based Corporate Network[J]. Environment & Planning: A: Economy and Space 49(12):2916-2937.

Snyder, D., and E. L. Kick. 1979. “Topological Position in the World System and Economic Growth, 1955-1970: A Multiple-Network Analysis of Transnational Interactions.” American

Journal of Sociology 84(5):1096-1126.

Watts, D. J., and S. H. Strogatz. 1998. “Collective Dynamics of ‘Small-World’ Networks.” Nature 393:409-410.

White, H. C., S. A. Boorman, and R. L. Breiger. 1976. “Social Structure from Multiple Networks I: Block Models of Roles and Positions.” American Journal of Sociology 81(4): 730-780.

Xi, N., R. Muneepeerakul, S. Azaele, and Y. G. Wang. 2014. “Maximum Entropy Model for Business Cycle Synchronization.” Physica A: Statistical Mechanics and its Applications 413:189- 194.

Yao, W., and A. D. Tang. 2020. “Financial Integration and Business Cycle Synchronization.” China Journal of Economics 2020,7(2):61-85. (In Chinese).
