# Supplementary material for: Topological characteristics of international business cycle synchronization: A network analysis of the BRI economies
Source: PLoS One. 2022 Jun 28;17(6):e0270333. doi: 10.1371/journal.pone.0270333 (PMC9239471; doi:10.1371/journal.pone.0270333)
Supplement: Attachment — Submitted filename: Response to Reviewers.docx [file pone.0270333.s006.docx]

**Responses to Reviewer #1:**

Dear reviewer:

Thank you for your comments on our manuscript. Those comments are valuable and very helpful for revising and improving our paper. We have studied the comments carefully and have made corrections which we hope to meet with approval finally. Below we response each issue that the reviewers raised, and describe corresponding changes by using **red** text in the revised manuscript.

Following you detailed comments in PDF manuscript, we improved the quality of this manuscript based on your constructive comments. Here we submitted a new version, which has been further revised according to your suggestions. In summary, major modifications include:

According to your advice, we have revised the abstract on page 1, footnote of introduction on page 3 and the format of references on page 25-29. In addition, we also checked the content of other parts of this paper and corrected the errors found.

In the end, it should also be noted that in the last paragraph of page 10 and the formula (2) on page 11, due to the mistake in the last revised manuscript, we misused the **row** **mean** of the matrix ${\sum_{\boldsymbol{j}}^{\boldsymbol{N}} \boldsymbol{S}_{\boldsymbol{ij}}}/\boldsymbol{N}$ as the threshold value for data processing. In fact, we should use the **mean** of the matrix ${\sum_{\boldsymbol{i}}^{\boldsymbol{N}} \sum_{\boldsymbol{j}}^{\boldsymbol{N}} \boldsymbol{S}_{\boldsymbol{ij}}}/\boldsymbol{N(N-1)}$ as the threshold value instead, and the undirected and weighted BCSN could be unweighted to obtain the adjacency matrix A composed of 0 and 1. In view of this, we have modified the relevant explanations in the last paragraph of page 10 and the formula (2) on page 11. The above modifications do not affect the reliability and validity of the empirical results of this paper.
